# Glucose-Coated Superparamagnetic Iron Oxide Nanoparticles Prepared by Metal Vapour Synthesis Are Electively Internalized in a Pancreatic Adenocarcinoma Cell Line Expressing GLUT1 Transporter

**DOI:** 10.1371/journal.pone.0123159

**Published:** 2015-04-15

**Authors:** Daniele Barbaro, Lorenzo Di Bari, Valentina Gandin, Claudio Evangelisti, Giovanni Vitulli, Eleonora Schiavi, Cristina Marzano, Anna M. Ferretti, Piero Salvadori

**Affiliations:** 1 Section of Endocrinology, General Hospital, Livorno, Viale Alfieri 36, 57100 Livorno, Italy; 2 Department of Chemistry and Industrial Chemistry, University of Pisa, Via Moruzzi 3, 56124 Pisa, Italy; 3 Department of Pharmaceutical and Pharmacological Sciences, University of Padova, Via F. Marzolo 5, 35100 Padova, Italy; 4 Institute of Molecular Science and Technologies, National Research Council, Via G. Fantoli 16/15, I-20138 Milano, Italy; 5 ErreDue SpA, Via G. Gozzano 3, 57121 Livorno, Italy; Institute for Materials Science, GERMANY

## Abstract

Iron oxide nanoparticles (IONP) can have a variety of biomedical applications due to their visualization properties through Magnetic Resonance Imaging (MRI) and heating with radio frequency or alternating magnetic fields. In the oncological field, coating IONP with organic compounds to provide specific features and to achieve the ability of binding specific molecular targets appears to be very promising. To take advantage of the high avidity of tumor cells for glucose, we report the development of very small glucose-coated IONP (glc-IONP) by employing an innovative technique, Metal Vapor Synthesis (MVS). Moreover, we tested the internalization of our gl-IONP on a tumor line, BxPC3, over-expressing GLUT 1 transporter. Both glc-IONP and polyvinylpyrrolidone-IONP (PVP-IONP), as control, were prepared with MVS and were tested on BxPC3 at various concentrations. To evaluate the role of GLUT-1 transporter, we also investigated the effect of adding a polyclonal anti-GLUT1 antibody. After proper treatment, the iron value was assessed by atomic absorption spectrometer, reported in mcg/L and expressed in mg of protein. Our IONP prepared with MVS were very small and homogeneously distributed in a narrow range (1.75-3.75 nm) with an average size of 2.7 nm and were super-paramagnetic. Glc-IONP were internalized by BxPC3 cells in a larger amount than PVP-IONP. After 6h of treatment with 50 mcg/mL of IONPs, the content of Fe was 1.5 times higher in glc-IONP-treated cells compared with PVP-IONP-treated cells. After 1h pre-treatment with anti-GLUT1, a reduction of 41% cellular accumulation of glc-IONP was observed. Conversely, the uptake of PVP-IONPs was reduced only by 14% with antibody pretreatment. In conclusion, MVS allowed us to prepare small, homogeneous, super-paramagnetic glc-IONP, which are electively internalized by a tumor line over-expressing GLUT1. Our glc-IONP appear to have many requisites for in vivo use.

## Introduction

Iron oxide nanoparticles (IONP) can have a variety of biomedical applications such as drug delivery, Magnetic Resonance Imaging (MRI) and endogenous hyperthermia by heating IONP with radio frequency or alternating magnetic fields [1–7]. Coating IONP with organic compounds to provide specific features and to achieve the ability of binding specific molecular targets represents one of the most promising fields of study [1–3]. The organic surface must be non-toxic, ensure stability and have bio and physico-chemical characteristics of good bio-compatibility [5]. Tumor cells have the ability to uptake dextrane-coated magnetite nanoparticles by non-specific endocytosis. Local injection directly into the tumor mass of IONP, coated with different polymers, has already been proved to be successful for the thermotherapy of various tumor types [8–16]. However, as stated above, a coating containing a ligand that can specifically target a tumor cell would appear more suitable, thus leading to a selective uptake and accumulation of IONP into tumor areas, allowing for intravenous systemic use. As is known, increased glucose uptake, mainly through glycolitic anaerobic pathway, is one of the earliest and well-recognized metabolic alterations in the transformed cell [23]. This anomaly, known as the Warburg effect, represents the rationale of Positron Emission Tomography (PET) using Fluorine-18-fluorodeoxyglucose (18-FDG), which, either alone or combined with computed tomography, has become a routine clinical test for the diagnosis and staging of cancer [17]. Many studies have actually demonstrated that the expression of glucose transporters, especially GLUT1, increases in a wide variety of malignancies. Moreover, GLUT1 overexpression has been found to be associated with tumor progression and with poor overall patient survival in various malignant tumors [23,24]. Therefore, GLUT1 could represent a useful way for transporting nanomolecules inside cancer cells.

Following these concepts, and with the aim of targeting GLUT-overexpressing cancer cells, some papers have reported on the development of 2-deoxy-glucose (2DG) coated IONP [18,19]. Based on the literature findings, the optimal features of glucose (or its analogues) coated IONP should: i) have good magnetic properties; ii) have a small hydrodynamic radius in order to facilitate penetration through capillary endothelium and distribution in the interstitial fluid; iii) have a narrow distribution of the iron oxide core around an “optimal” value. Despite the difficulty of establishing the optimal small size and a minimum ratio between the inorganic and organic components this can allow for more physiological transport inside the cells. On the other hand, as IONP that are too small may not display the desired magnetic properties, a middle ground must be found.

To this end, we addressed a less common way of obtaining metal nanoparticles called Metal Vapor Synthesis (MVS) [20–22]. This technique has at least two notable advantages which are particularly relevant in the development of materials to be used in biomedicine. First, it allows small and homogeneous metal nanoparticles to be produced and second, the use of reactants during the nanoparticles production can be avoided. This is because it is based on the simple sublimation/recondensation of the metal under high vacuum. Using MVS we have prepared small D-glucose-coated IONP (glc-IONP) which display useful magnetic properties. Glc-IONP have been characterized by their morphological and magnetic properties, and were tested for their ability to accumulate in human pancreatic cancer cells expressing cell membrane glucose transporter GLUT-1.

## Results

### Characterization of IONP

**Fig 1:**
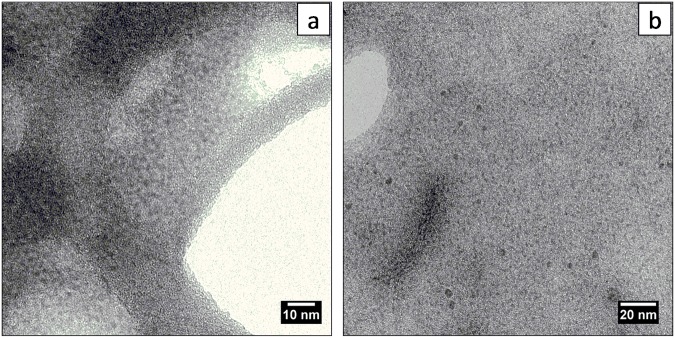
TEM micrographs: a) glc-IONP (magnification 250000x); b) PVP-IONP (magnification 160000x).

TEM and STEM analysis of the Fe_x_O_y_-glc system revealed, as shown in Fig 1, the presence of very small metal nanoparticles, homogeneously populated and mainly distributed in a narrow range (1.75 nm–3.75 nm) with a mean diameter of 2.7 nm.

**Fig 2:**
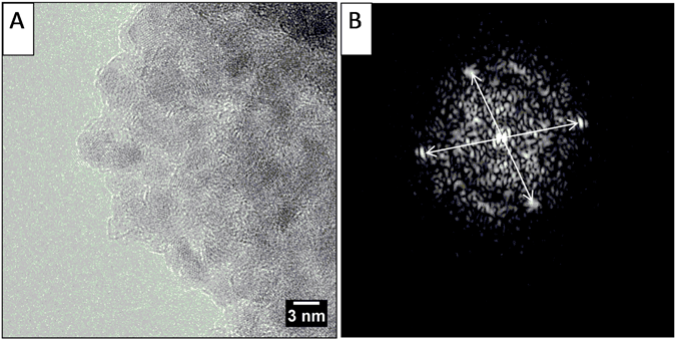
High Resolution TEM micrograph, magnification 600000x (A) and FFT image performed on a particle of glc-IONP (B).

In order to investigate the iron oxide formed during the oxidation process, a lattice fringe analysis on the HRTEM images was performed (Fig 2). Lattice fringe analysis recorded on larger iron oxide particles exhibits spots in the FFT (Fast Fourier Transform) pattern at 2.5 Å and 2.4 Å. These can be ascribed to the spacing of (3 1 1) and (2 2 2) planes of spinel structure of maghemite (γ-Fe_2_O_3_) crystal, where iron atoms are completely oxidated to Fe(III). However, considering the particularly small size of the particles, the low presence of iron oxide in the magnetite (Fe_3_O_4_) crystal structure cannot be completely excluded.

**Fig 3:**
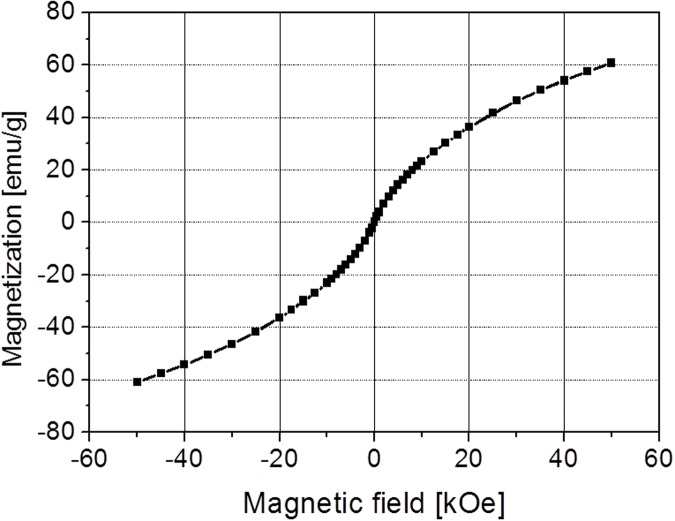
Magnetization (emu·g^-1^) vs. applied field (Oe) at 4 K.

Fig 3 shows the magnetization, expressed in emu/g, of material as a function of the applied field. The loop does not show hysteresis which means Fe_x_O_y_-glc nanoparticles are superparamagnetic not only at room temperature but also at T = 5 K. The maximum value of magnetization, measured at 50k Oe, is 60.75 emu/g, which is not yet the saturation value. This result is evidence that the studied sample is in a superparamagnetic regime also at T = 5 K.

The relaxivities measured at 7 T resulted R_1_ = 0.1 s^-1^·mM(Fe)^-1^ and R_2_ = 23.3 s^-1^·mM(Fe)^-1^.

### Expression of GLUT1 in BxPC3 cells

Since the aim of our study was to determine whether glc-IONP cellular accumulation is mediated by glucose transporters, such as GLUT1, we assessed its expression levels in pancreatic adenocarcinoma BxPC3 cells and in normal MRC5 lung cells expressing low levels of GLUT1.

**Fig 4:**
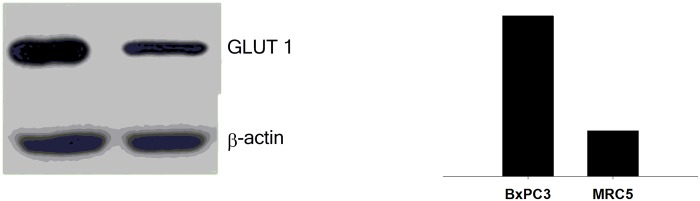
GLUT1 levels estimated by Western blotting in BxPC3 and MRC5 cells. The immunoblot (left panel) bands were analyzed (right panel) for the intensity of the areas by OD with Image J software.

Based on data found in the literature, the Western blot results reported in Fig 4 clearly indicate that BxPC3 cells posses a superior level of GLUT1 with respect to MRC5 cells. Moreover, densitometric analysis revealed that GLUT1 was 3.7-fold higher in cancer cells than in non-transformed cells.

### Cytotoxicity tests

**Fig 5:**
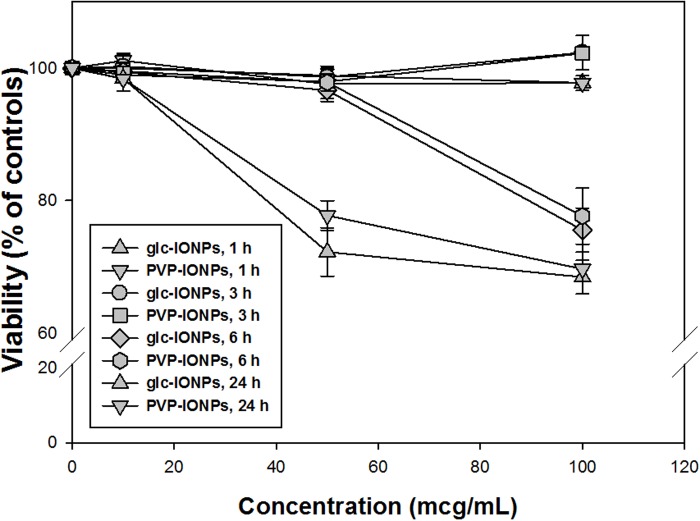
Sensitivity of BxPC3 cells to glc-IONPs and PVP-IONPs. Cells were treated for 1, 3, 6 and 24h with increasing concentrations. IONPs and cell viability was determined by MTT assay. Values are the mean (±SD) of three independent experiments.

The results regarding cytotoxicity are reported in Fig 5. Neither of the IONPs at the lowest concentration (10 mcg/mL) determined a significant reduction of cell viability following all the tested exposure times. Similarly, no cytotoxic effects were detected in BxPC3 treated for 1, 3 and 6h with 50 mcg/mL of glc-IONP and polyvinylpyrrolidone IONP (PVP-IONP). However, a slight increase in cell-killing ability was seen after 24h of treatment with 50 mcg/mL of both IONPs with a reduction of BxPC3 cell viability of 28% and 22% respectively. At the highest concentration (100 mcg/mL), a substantial loss of cell viability was noted following 6 and 24h exposure with glc-IONP and PVP-IONP (31% and 30%, respectively).

### Cellular uptake

Based on cytotoxicity data, the cellular uptake of glc-IONP and PVP-IONP was evaluated using concentrations and time exposures that did not affect cell viability. In particular, 10 and 50 mcg/mL for 1, 3 and 6h exposure times were chosen as appropriate treatment conditions. BxPC3 were treated with glc-IONP and PVP-IONP, and Fe content was determined by GF-AAS. The results, expressed as ng/L/mg of proteins and reported in S 6 (panel A and B), show a time and concentration dependent internalization of our IONP (p<0.001). But, as shown again in Fig 5, glc-IONP are internalized by BxPC3 cells in a larger amount than PVP-IONP (p <0.001). In particular, after 6h of treatment with 50 mcg/mL of IONPs, the content of Fe was 1.5 times higher in glc-IONP-treated cells compared with PVP-IONP-treated cells. Moreover, notwithstanding that the uptake of both IONPs looks firmly time-dependent, a different behavior can be perceived concerning dose-dependency: PVP-IONPs uptake follows a linear dose-dependence, whereas the uptake of glc-IONP appears to follow a time-dependent kinetic saturation, a typical feature of carrier-assisted cellular internalization.

In our attempt to assess whether glc-IONP uptake is mediated by GLUT1, we performed uptake experiments in BxPC3 cells pretreated with polyclonal anti-GLUT1.

**Fig 6:**
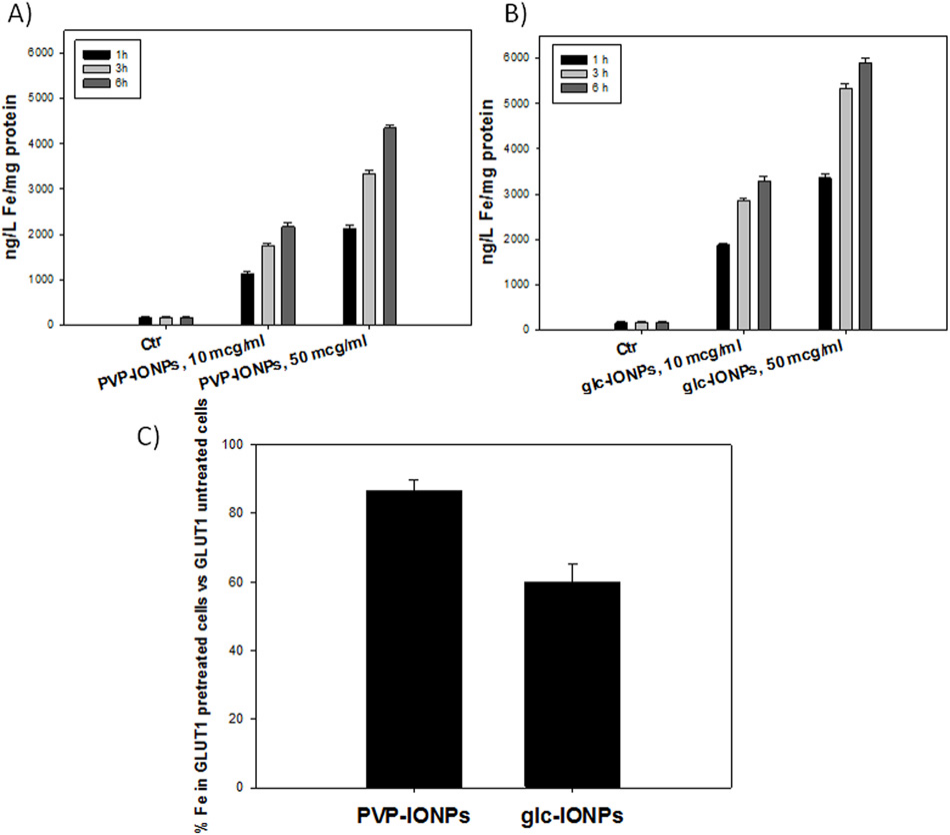
BxPC3 cells were incubated with 10 and 50 mcg/mL of glc-IONP (panel A) and PVP-IONP (panel B) for 1, 3 or 6h. The Fe cellular content was estimated by means of GF-AAS analysis. Panel C represents the percentage of inhibition of internalization of PVP-IONP and glc-IONP after pre-tratment with anti GLU1 antibody. Values are the mean (±SD) of three independent experiments.

After 1h pretreatment with anti-GLUT1, a reduction of 41% in cellular accumulation of glc-IONPs was observed (Fig 6, panel C). Conversely, the uptake of PVP-IONPs was reduced only by 14% with antibody pretreatment (p <0.001). Taken together, these uptake data suggest the involvement of carrier-mediated cellular internalization for glc-IONPs, whereas no indication of facilitated transport across plasma membrane can be supposed in the case of PVP-IONPs.

### IONP cellular localization

**Fig 7:**
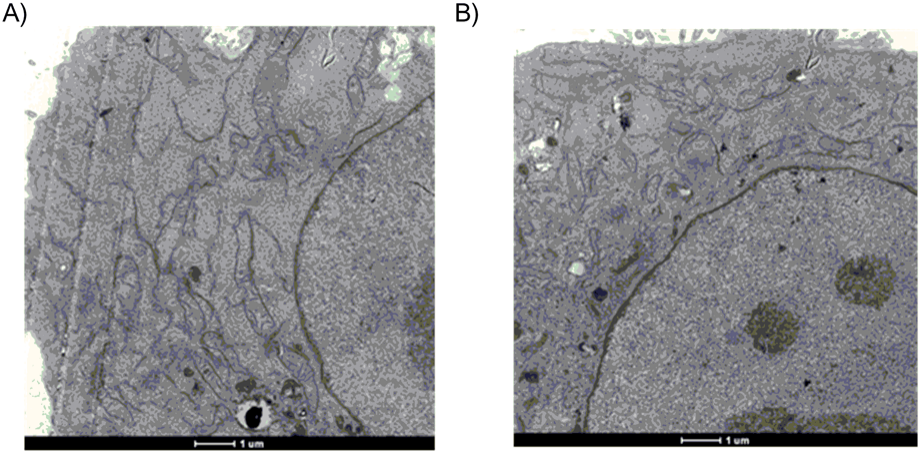
TEM images at 20 nm zoom of: a) glc-IONP-treated cells; b) PVP-IONP-treated cells.

TEM analysis of BxPC3 cells treated with IONP were performed to gain insights into the cellular distribution of IONP. Fig 7 reports the results after treatment for 6h with 50 mcg/mL which, as already demonstrated, gave the highest concentration inside the cells without significant cytotoxicity.

TEM analysis was difficult due to the particularly small size of our IONP and only clusters were in fact well visible. However, as represented in Fig 7, TEM revealed that the visible glc-IONP were localized in vesicles confined to the cytosol, and no glc-IONP were detected within cellular organelles or in the nucleus. On the other hand, besides localizing in the cytosol, PVP-IONP appeared to be able to reach intranuclear regions. TEM analysis performed after a longer time or higher concentration showed the same different localization inside the cells for both IONP but, according to MTT assay, also confirmed degenerative cell phenomena.

## Discussion

One of the most widely documented metabolic activities in tumors is the so-called Warburg effect named after Otto Warburg who, in the 1920s, observed that tumor cells consume a large amount of glucose, much more than normal cells, and convert most of it to lactic acid [23]. In the modern era, gene analysis has demonstrated the nearly ubiquitous over-expression of glycolitic genes and GLUT transporter, mainly GLUT 1 and/or GLUT 4, across a wide spectrum of human cancers [23–25].

In the oncologic field, using the analogue of glucose, 2DG, as a vector to target GLUT-overexpressing cells with radioactive tracer, already finds wide clinical application with ^18^F-FDG PET. Using glucose as a coating for nanoparticles could represent a Trojan horse to deliver IONP inside the tumor cells of a wide variety of malignancies. A fundamental pre-requisite is that the glucose molecules covering IONP remain intact in order to be recognized by their specific transporter. In fact, cellular uptake can be GLUT1-mediated if the hydroxyl groups of glucose molecule, which possess a high affinity toward GLUTI 1, are available and correctly oriented in order to be involved in hydrogen binding with the transmembrane alpha helices of GLUT 1 [27]. Our data show that glc-IONP are internalized inside the cells in a larger amount than PVP-IONP. The fact that PVP-IONP can also enter the cell is not surprising because, as is known, tumor cells have the ability to pinocyte NP of different natures, and the difference in uptake of glc-IONP vs. PVP-IONP can represent the amount of glc-IONP using the GLUT transporter. These results are also in agreement with two recent papers, demonstrating that 2-Deoxy-D-Glucose functionalized IONP were electively internalized in tumor lines [18–19]. However, in one of these papers the analysis was performed by a qualitative method (optical microscope and TEM observations), while in the other paper a quantitative UV colorimetric assay was employed. In this paper, we demonstrate that our glc-IONP are internalized by TEM and we also provide quantitative data by means of ICP-AA normalized to total protein content of the samples.

To gain further insight, we proceeded to variable dose and exposure time experiments. These data demonstrate that the uptake of glc-IONP follows a mechanism which is different from PVP-IONP: the former displays a time-dependent saturation kinetic as is expected from a carrier-mediated process. In contrast, PVP-IONP uptake shows a linear kinetic more typical of a non-specific endocytosis pathway. [26,28]

This behavior of our glc-IONP may be due to the particularly small iron cores of our NP and this can explain the difference with respect to previous data [19], which display a linear kinetic of 2DG internalization.

The anti-GLUT1 antibody experiments confirm the role of GLUT1 transporter; we find a remarkable inhibition of glc-IONP uptake, while PVP-IONP show only a slight decrease in internalization. The fact that PVP-IONP also appear to have some inhibition of the uptake can be due to a generic interference either with the binding of anti-GLUT1 to the membrane or on the cell metabolism.

At this stage, it is impossible to determine what the exact uptake mechanism might be and if other GLUTs, other than GLUT1, expressed on the cell membranes could contribute. As already stated, the observed time-dependent saturation kinetic suggests a mechanism based on facilitated transport. It is however impossible to say if it is endocytosis receptor-mediated or some other mechanism. In fact, our IONP are particularly small and we can therefore suppose that they pass across the membrane by non-vescicular transport, as described for small glucose-coated gold nanoparticles [29].

A potential drawback for in vivo use of such small IONP could be fast leakage from the vasculature into normal tissue, and the difficulty of achieving optimal concentration in tumor tissue. On the other hand, they can be cleared by the kidneys, with low body accumulation and minor risk of toxicity [30–32].

To translate these data into a potential field of development in vivo we would need to have homogeneous NP, preparation facility, absence of contaminants and optimal magnetic properties.

We prepared IONP by means of the Metal Vapor Synthesis (MVS) protocol, which appears to guarantee all these features. In fact, during the iron clusterization phase, a precise control of the experimental parameters allows us to obtain small and homogeneous Fe nanoparticles. The subsequent oxidation with O_2_ and treatment with a glucose syrup (or a PVP solution) yields glucose (or PVP) coated iron oxide nanoparticles, which are precipitated and recovered. In doing this no redox reactant (apart from O_2_) is used, which renders isolation and purification of the IONP particularly simple. Moreover, only residual solvent (acetone) removal by evaporation is required, and this ensures absence of contaminants and a clean procedure. As already stated in the results, characterization of the IONP by electron microscopy (HR-TEM) reveals that they are monodispersed, with a mean diameter of 2.7 and that, despite being very small, they are superparamagnetic and display good properties. The last point to be examined is the adhesion of glucose and PVP to the iron oxide core. It appears strong because IONP are washed several times until organic molecules are no longer released but we don’t know the nature of this interaction. A further characterization and study of our IONP will be necessary but we believe that, at this stage, such a study would go beyond the scope of the present investigation.

In conclusion, MVS has allowed us to prepare small, homogeneous, superparamagnetic glc-IONP, which are electively internalized by a line of tumor cells expressing GLUT 1. Further experiments in vitro are needed to clarify the mechanism of uptake, and further tests on cell lines with different expression of other GLUT transporters will help us to realize if our glc-IONP are specifically recognized by some and not others. However, good biocompatibility, absence of contaminants and evidence of internalization of our glc-IONP are basic requisites for use in vivo. The superparamagnetic properties can be suitable both for MRI and therapeutic hyperthermia.

## Materials and Methods

### Preparation of IONP

The co-condensation of iron and acetone vapor was carried out in a static reactor, described elsewhere [16–18], and equipped with an alumina-coated tungsten crucible heated by Joule effect with a generator with a maximum power of 2 kW. The solvated metal atom (SMA) solutions were handled under argon atmosphere with the use of the standard Schlenk techniques.

Acetone (Aldrich) was distilled over KMnO_4_ and stored under argon before use. D-Glucose was purchased from Aldrich and used without further purification. The amount of iron in a solution was determined by a Spectro Genesis Inductively Coupled Plasma-Optical Emission Spectrometer (ICP-OES). For ICP-OES, a sample (1 mL) of SMA solution was heated three separate times over a heating plate in a porcelain crucible in the presence of aqua regia (2 mL), dissolving the solid residue in 0.5 M aqueous HCl. Fe vapors were generated by resistive heating of an alumina-covered tungsten crucible filled with Fe chips (300 mg) and co-condensed with acetone (100 mL) at the liquid nitrogen temperature on the reactor walls. The reactor chamber was warmed at the melting point of the solid matrix (-80°C) and the resulting brown solution of Fe/acetone was siphoned and kept at low temperature (-40°C). The iron content, obtained by ICP-OES analysis, was 1.55 mg/mL.

### Preparation of glc-IONP

**Fig 8:**
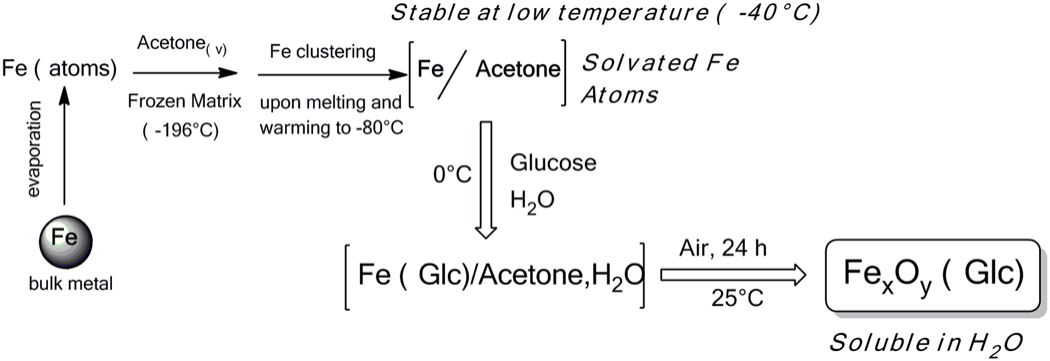
Preparation of Glucose-coated iron nanoparticles using our metal vapor synthesis protocol.

As depicted in Fig 8, portion of the Fe/acetone solution (50 mL, 1.39 mmols of Fe) was added to an aqueous solution of D-Glucose (5.00 g in 15 mL H_2_O) kept at 0°C inside a Schlenk tube (100 mL). The dispersion was warmed up to room temperature (25°C) by gentle stirring at air temperature for 24 hours.

The obtained precipitate was further decanted, filtered with a Buchner funnel and washed three times with water (5 mL) and acetone (5 mL) respectively. This allowed 1.9 g of the FexOy-D-Glucose system containing 2.3 wt.% of Fe (ICP-OES analysis) to be obtained.

### Preparation of PVP-IONP

A portion of the Fe/acetone solution (100 mL, 2.78 mmols of Fe) was added to 60 mL of an ethanol solution of polyvinylpyrrolidone K25 (Fluka) (10.00 g in 60 mL EtOH) kept at 0°C inside a Schlenk tube at 0°C for 2h under O_2_ atmosphere. The dispersion was warmed up to room temperature (25°C) and ethyl ether was added (1L) to precipitate an orange colloid, which was washed with acetone and ether (3 times each, 10 mL each) before drying under vacuum. The collected solid contained 2.0 wt.% of Fe (ICP-OES analysis).

### Analysis by transmission electron microscope (TEM)

Electron micrographs were obtained by a Zeiss LIBRA 200FE analytical transmission electron microscope (TEM) equipped with: 200 kV FEG, in column second-generation omega filter for energy selective spectroscopy (EELS) and imaging (ESI), HAADF STEM facility, EDS probe for chemical analysis, integrated tomographic HW and SW system. Before the introduction into the instrument, the samples, in the form of powders, were ultrasonically solubilized in water and a drop of the solution was deposited on a holey-carbon film supported on a copper TEM grid 300 mesh. Histograms of the particle size distribution were obtained by counting at least 500 particles on the micrographs; the mean particle diameter (dm) was calculated by using the formula dm = Σd_i_n_i_/Σn_i_, where n_i_ was the number of particles of diameter d_i_.

### Analysis of magnetic properties

For the measurement of magnetic properties a weighted amount of precipitated and dried NPs was packaged in Teflon tape. Hysteresis loop was measured at 5 K after field cooling at 50 kOe. Magnetic measurements were carried out by a Quantum Design MPMS-5 SQUID magnetometer.

### Relaxivity at 7T

Longitudinal and transverse relaxivities of glc-IONP where measured on a Varian VXR 300 NMR spectrometer operating at 7T. Five water solutions at variable concentrations of IONP between 0.5 and 2 mg of glucose-coated IONP (equivalent to 11 to 43μg of Fe) were measured (linewidth afforded an upper limit for transverse relaxation rate ρ_2_, and inversion recovery provided the longitudinal rate ρ_1_). Linear interpolation (R>0.99) of ρ_1_ or ρ_2_ vs. [Fe] concentration afforded the longitudinal and transverse relaxivities.

### Cell cultures

GLUT1-expressing BxPC3 cells, derived from a human pancreatic ductal adenocarcinoma, were obtained from the American Type Culture Collection (ATCC; Manassas VA). BxPC3 were grown at 37°C in a 5% carbon dioxide atmosphere and cultured in RPMI 1640 with 2mM Glutamine, 10% Foetal Bovine Serum (FBS) and supplemented with 50 units ml^-1^ penicillin and 50 μg ml^-1^ streptomycin.

### Cytotoxicity assays

The growth inhibitory effect toward BxPC3 cells was evaluated by means of the MTT assay.5·10^3^ cells well^-1^ were seeded in 96-well microplates in growth medium (100 μL) and then incubated at 37°C in a 5% CO_2_ atmosphere. After 24h the medium was removed and replaced with a fresh one containing the IONP. Cells were treated for different time exposures (1, 3, 6 and 24h) with increasing concentrations (10, 50 and 100 mcg/mL) of glc-IONP and PVP-IONP dissolved in 0.9% NaCl solution just before use. Triplicate cultures were established for each treatment. After exposure times, each well was treated with 10 μL of a 5mg·mL^-1^ MTT saline solution and after 5h of incubation, 100 μL of a sodium dodecyl sulfate (SDS) solution in HCl 0.01M was added. After overnight incubation, the inhibition of cell growth induced by tested IONP was determined by measuring the absorbance of each well at 570 nm, using a Bio-Rad 680 microplate reader. Mean absorbance for each IONP concentration was expressed as percentage of the control and plotted versus IONP concentration. The final value is the mean ± S.D. of at least three independent experiments performed in triplicate.

### Evaluation of GLUT-1 expression on BxPC3 cells

Western blot analyses: About 10^6^ BxPC3 cells were harvested and lysed in RIPA buffer (1% NP40, 0.5% sodium deoxycholate, 0.1% SDS) and centrifuged at 13,000xg for 15 min at 4°C. β-actin was used as a loading control. An equal amount of proteins for each sample was electrophoresed on a 12% SDS-PAGE, and blotted to a nitrocellulose membrane. The membrane was incubated for 1h in PBS-Tween20 (0.05%), containing 5% non-fat milk, and then at 37°C for 1h with primary antibodies, namely rabbit anti-human β-actin polyclonal and rabbit anti-human Glut 1 polyclonal antibodies purchased from Abcam (Cambridge, UK). The membranes were stained with the corresponding peroxidase-conjugated secondary antibodies for 1h at room temperature, and detected by ECL according to the manufacturer’s protocol (GE Healthcare, Fairfield, CT).

### Evaluation of Fe uptake

Quantitative analysis: BxPC3 cells (2.5·106) were seeded in 75 cm2 flasks in growth medium (20 mL). After 24h the medium was replaced and the cells were incubated for 1, 2 and 6h in the presence of 0.01 and 0.05 mg/mL of the tested IONP. Cell monolayers were washed twice with cold PBS (2 mL) and harvested. Samples were subjected to three freeze/thaw cycles at -80°C and then vigorously vortexed. Aliquots were removed for the determination of protein content by the Bradford protein assay (BioRad). The samples were treated with 1 mL of highly pure nitric acid (Fe: <0.01 mg·kg-1, TraceSELECT Ultra, Sigma Chemical Co.) and transferred into a microwave Teflon vessel. Subsequently, samples were submitted to standard procedure using a speed wave MWS-3 Berghof instrument (Eningen, Germany). After cooling, each mineralized sample was analyzed for iron by using a Varian AA Duo graphite furnace atomic absorption spectrometer (Varian, Palo Alto, CA, USA) at 248.3 nm. The iron value was reported in mcg/L and expressed in mg of protein. Each experiment was repeated in triplicate and values reported as mean +/- SD. Statistical analysis was performed with StatView software, version 5.0.11 (abacus Concepts, Inc, Berkeley, CA). A value of p<0.005 was considered statistically significant.

Statistical comparison between PVP-IONP and Glc-IONP for each concentration and time was performed with t-Student test while ANOVA was used for comparison at the various times for each concentration for both nanoperticles.

The calibration curve was obtained using known concentrations of standard solutions purchased from Sigma Chemical Co.

In competition uptake experiments, cells were pre-incubated with anti-GLUT1 polyclonal antibody (Abcam, Cambridge, UK) for 30 min before exposure to tested IONPs. The statistical comparison was performed using the same software by t-student test.

Transmission electron microscopy analyses: About 10^6^ BxPC3 cells were seeded in 10 cm petri dishes. After 24h the medium was removed and replaced with a fresh one containing the IONP. Cells were treated for different time exposures (1, 3, 6 and 24h) with increasing concentrations (10, 50 and 100 mcg/mL) of glc-IONP and PVP-IONP. Subsequently, cells were washed in cold PBS, harvested and directly fixed in 1.5% glutaraldehyde buffered with 0.2 M sodium cacodylate, pH 7.4. After washing in the buffer and postfixation in 1% OsO_4_ in 0.2 M cacodylate buffer, specimens were dehydrated and embedded in epoxy resin (Epon Araldite). Sagittal serial sections (1 μm) were counterstained with toluidine blue; thin sections (90 nm) were given a contrast by staining with uranyl acetate and lead citrate. Micrographs were taken with a Hitachi H-600 electron microscope (Hitachi, Tokyo, Japan) operating at 75 kV. All photos were typeset in Corel Draw 11.
